# A Moderate Blast Exposure Results in Dysregulated Gene Network Activity Related to Cell Death, Survival, Structure, and Metabolism

**DOI:** 10.3389/fneur.2020.00091

**Published:** 2020-02-26

**Authors:** Katie A. Edwards, Vida Motamedi, Nicole D. Osier, Hyung-Suk Kim, Sijung Yun, Young-Eun Cho, Chen Lai, Kristine C. Dell, Walter Carr, Peter Walker, Stephen Ahlers, Matthew LoPresti, Angela Yarnell, Anna Tschiffley, Jessica M. Gill

**Affiliations:** ^1^National Institute of Nursing Research, National Institutes of Health, Bethesda, MD, United States; ^2^The Henry M. Jackson Foundation for the Advancement of Military Medicine, Bethesda, MD, United States; ^3^Wake Forest School of Medicine, Wake Forest University, Winston-Salem, NC, United States; ^4^School of Nursing, University of Texas at Austin, Austin, TX, United States; ^5^Department of Neurology, University of Texas, Austin, TX, United States; ^6^College of Nursing, University of Iowa, Iowa City, IA, United States; ^7^Walter Reed Army Institute of Research, Silver Spring, MD, United States; ^8^Naval Medical Research Center, Silver Spring, MD, United States; ^9^CNRM Co-Director Biomarkers Core, Uniformed Services University of the Health Sciences, Bethesda, MD, United States

**Keywords:** blast, overpressure, gene expression, RNA-sequencing, NanoString

## Abstract

Blast exposure is common in military personnel during training and combat operations, yet biological mechanisms related to cell survival and function that coordinate recovery remain poorly understood. This study explored how moderate blast exposure influences gene expression; specifically, gene-network changes following moderate blast exposure. On day 1 (baseline) of a 10-day military training program, blood samples were drawn, and health and demographic information collected. Helmets equipped with bilateral sensors worn throughout training measured overpressure in pounds per square inch (psi). On day 7, some participants experienced moderate blast exposure (peak pressure ≥5 psi). On day 10, 3 days post-exposure, blood was collected and compared to baseline with RNA-sequencing to establish gene expression changes. Based on dysregulation data from RNA-sequencing, followed by top gene networks identified with Ingenuity Pathway Analysis, a subset of genes was validated (NanoString). Five gene networks were dysregulated; specifically, two highly significant networks: (1) Cell Death and Survival (score: 42), including 70 genes, with 50 downregulated and (2) Cell Structure, Function, and Metabolism (score: 41), including 69 genes, with 41 downregulated. Genes related to ubiquitination, including neuronal development and repair: UPF1, RNA Helicase and ATPase (*UPF1*) was upregulated while UPF3 Regulator of Nonsense Transcripts Homolog B (*UPF3B*) was downregulated. Genes related to inflammation were upregulated, including AKT serine/threonine kinase 1 (*AKT1*), a gene coordinating cellular recovery following TBIs. Moderate blast exposure induced significant gene expression changes including gene networks involved in (1) cell death and survival and (2) cellular development and function. The present findings may have implications for understanding blast exposure pathology and subsequent recovery efforts.

## Introduction

When an individual is in close proximity to a blast, the resulting overpressure (i.e., shock wave) can cause injury to the brain and/or body (1). The increased use of improvised explosives, sophisticated weaponry, and explosive entry techniques has led to increased risk of blast exposure. Specifically, in military personnel who deployed to recent conflicts of Operation Iraqi Freedom (3) and Operation Enduring Freedom (OEF), an estimated 300,000 service members were exposed to at least one blast from adversary attack (2), and blast overpressure from firing weapons is increasing commensurate with increases in weaponry power. High intensity blast exposure events can damage connective tissues, including the central nervous system, resulting in cerebrovascular damage and blood-brain barrier disruption. Significant overpressure can result in tearing of the long axons of neurons (diffuse axonal injury) leading to the associated deficits and comorbidities of a traumatic brain injury (TBI) (4, 5). Although there is evidence suggesting blast-induced TBI (biTBI) has distinct features from blunt-force or penetrating TBI (6), it is difficult to evaluate the consequences of blast in isolation using human subjects because there is often concomitant blunt force or penetrating TBI when objects are propelled and contact the skull (e.g., shrapnel) or the individual is thrown. These challenges contribute to the relatively poor understanding of the pathophysiologic responses to blast and lack of therapies to treat blast-exposed individuals. Moreover, the response and subsequent recovery from blast exposure represent an important line of research, which remains to be further explored and may elucidate the biological mechanisms associated with blast.

Differential gene expression is reported in a small number of clinical TBI studies (7–10), with few studies relevant to blast TBI (11–13). Gene expression regulation is imperative to appropriate cellular response to external mechanical, environmental, or biological stimuli, and the nuclear factor kappa-light-chain enhancer of activated B cells (NF-κB) complex is a main transcription factor of these adaptive gene expression changes (14). More specifically, the NF-κB complex is a transcription factor central to numerous cellular pathways influencing cell survival and proliferation, including inflammatory and immune responses, gene activation, and ubiquitination (14). Animal models demonstrate that the NF-κB complex regulates the innate immune response through upregulation of proinflammatory cytokines including tumor necrosis factor (TNF) (15), interleukin 1 (IL-1) (16), and interleukin 6 (IL-6) (17). In addition, mutations and epigenetic changes within the NF-κB pathway have been linked to immune and inflammatory diseases (18). Cytokines are among a number of factors that may activate NF-κB. NF-κB becomes activated when ubiquitin degrades its inhibitory protein, IκK, freeing NF-κB to enter the nucleus and activate gene transcription (17, 19). Study of gene expression changes following blast exposure may elucidate some of these complexities surrounding the roles and relationships of ubiquitin and inflammatory cytokines following blast exposure.

Within clinical studies of TBI, changes in the NF-κB network are reported in a limited number of studies (7–10); however, they have not yet been examined in biTBI. Preclinical studies of blast exposures have demonstrated altered gene expression, including cognitive impairment (20, 21) and immune function (22). Recent work in military training that involves personnel exposure to blast has demonstrated that ubiquitin carboxy-terminal hydrolase-1 (UCH-L1) is weakly correlated with repeated exposure to low-level blast (12), which is consistent with previous work in TBI (23) and blast exposure (11, 24). In particular, Heinzelmann et al. (11) found protein ubiquitination genes (associated with neuronal recovery, central regulator in IPA) to be downregulated in military personnel with chronic symptoms following blast head injury. UCH-L1 is predominately expressed in the neurons and neuroendocrine cells within the brain (25, 26) and is an enzyme responsible for protein degradation, thus providing a role in ubiquitin stability within neurons and maintaining neuronal health (27). In animal models, a mutation in the UCH-L1 gene causing a truncated protein is associated with neurodegeneration, likely due to the buildup of ubiquitin and subsequent lack of protein clearance (28). Given this limited number of clinical studies, this study sought to further examine differential gene expression pathways in a blast-exposed population. The purpose of this study was to examine gene networks involving cell death and survival as well as cell structure, function, and metabolism to investigate the role of these networks specific to biTBI.

## Materials and Methods

To address the gaps in knowledge surrounding the consequences of exposure to isolated blast, a unique cohort of military personnel engaged in training on advanced techniques for breaching buildings with controlled explosives was utilized. The breaching activities were conducted under close supervision and with personal protective equipment and established safety procedures, eliminating the chance of concomitant blunt-force or penetrating TBI. Moreover, recruiting from a training environment (as opposed to real-world combat) facilitated accurate measurement of isolated blast exposures using helmets equipped with pressure sensors (see “Blast Measurement”). This novel sampling also facilitated a collection of baseline data, including pre-exposure blood draws to support assessment of gene expression changes after blast. During the 2 week training program, some participants (*n* = 29) experienced a moderate blast exposure with peak pressure exceeding 5 pounds per square inch (psi), which exceeded the training range limit of 4 psi and was more than 200% greater than typical exposures measured in such training [e.g., Carr et al. (12)]. The moderate blast exposure was an isolated event, and blast exposures remained ≤2 psi on all other training days. These 29 cases were studied for gene expression changes related to cell death and survival as well as cell structure, function, and metabolism from training day 1 to 10. Unbiased RNA-sequencing (RNA-seq) was used to detect dysregulated genes (13). Ingenuity pathway analysis (29) of dysregulated genes was used to identify gene networks, two of which were validated in the present study using NanoString's nCounter system.

### Participants

All study protocols were reviewed and approved by the Institutional Review Boards (IRBs) at the Naval Medical Research Center and Walter Reed Army Institute of Research (NMRC#2011.0002; WRAIR#1796) as described in a past publication (12). Prior to study participation, each participant provided informed consent. The parent study from which the present study is drawn was comprised of (*N* = 108) male active-duty military service members who were engaged in 2 week blast training programs (as either a student or instructor). The goal of the course was to teach advanced techniques for explosive breaching, a tactic used to gain access into secured structures. All participants provided demographic and health history data at baseline, as well as blood samples. For the present study, participants (*n* = 29) examined were those who experienced a moderate blast exposure (≥5 psi). These 29 individuals, who provided blood samples at the end of training (day 10), were used in the present study to examine gene expression changes from baseline to 3 days post-moderate blast exposure.

Self-reported data were provided by participants at baseline included demographic, health, and blast-history information. Demographic data included age, military rank, and educational status; health information collected included smoking status and history of TBI (Table 1). Previous blast exposure data were also obtained through self-reports on how many blast exposures had been experienced during breaching and artillery fires using the following ordinal scale: 0, 1–9, 10–39, 40–99, 100–199, 200–399, and 400+ blast exposures. Details regarding the surveys used to collect data have been previously described (12).

**Table 1:** Demographic and previous explosive exposure of participants exposed to moderate blast.

	**Moderate blast *(N* = 29)**
Mean age in years (SD)	31.2 (4.4)
Mean Years of Service (SD)	11.2 (4.7)
Number of Prior Explosive Breaches and Artillery	
Fires, % (no.)	
0–9	20.7% (6)
10–39	34.8% (10)
40–99	17.2% (5)
100–199	20.6% (6)
200–399	6.9% (2)

### Blast Measurement

Objective blast data were collected using standard Army combat helmets equipped with bilateral sensors capable of measuring blast parameters greater than a threshold of 0.4 psi on either sensor. Helmets were worn throughout training and the average of the right and left sensors was used as data to approximate levels of explosive blast each participant experienced. The sensitivity of the sensors is based on the technological specifications of the device itself (micro Data Acquisition System, μDAS; Applied Research Associates, Inc., Albuquerque, NM) as well as considerations for signal-to-noise ratios and effects on data interpretation.

### Laboratory Methods

#### Blood Sampling

Blood samples were collected at baseline and at the end of 2 week training, which was 3 days after moderate blast. Blood was collected in PAXgene tubes and stored in a −80°C freezer until the time of batch processing.

#### RNA-Sequencing

Random fragmentation of complementary deoxyribonucleic acid (cDNA) followed by 5′ and 3′ adapter ligation was used to create a cDNA library. Average fragment length was 150–170 bp. RNA integrity was assessed using Aligent Technologies 2100 Bioanalyzer and the mean value was 8.9 with standard error of 0.05. Samples from 29 participants on day 1 and 10 were sequenced for mRNA using the Illumina HiSeq-2500 Next Generation Sequencing system (Illumina Inc., San Diego, CA). Using this system, we performed RNA-seq to read paired-ends and read 101 bases per each end. Sequencing data used in the study were deposited in the Gene Expression Omnibus (GEO) with GEO ID GSE89866.

#### Ingenuity Pathway Analysis

**Figure 1:**
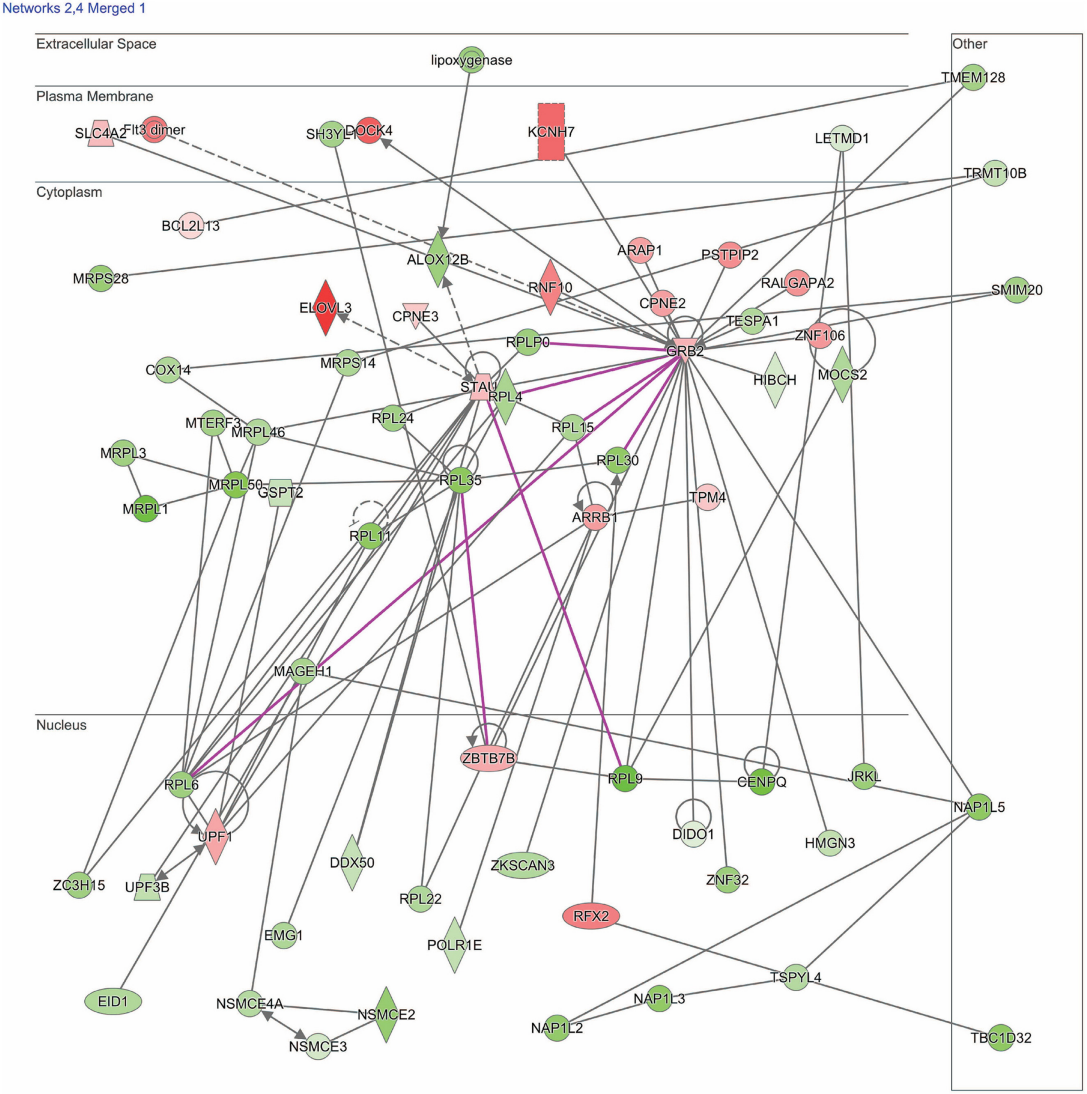
Network shows dysregulated cell death and survival pathway following moderate blast. Genes described in the text included: *UPF1, UPF3B, ARRB1, ZBTB7B, flt3, HIBCH, RPL6, RPL35, MRPL1, MRPL3, MRPL36*, and *MRPL50*. Red indicates increased measurement; green represents decreased measurement; with increased color saturation representing more extreme measurement in dataset. Solid lines represent direct interactions, non-targeting interactions, or correlations between chemicals, proteins, or RNA. Dotted lines represent indirect interactions. Purple lines denote the commonly found relationship among the two merged networks. Arrowed lines represent activation, causation, expression, localization, membership, modification, molecular cleavage, phosphorylation, protein-DNA interactions, protein-RNA interactions, regulation of binding, transcription. Shapes represent molecule type (double circle, complex/group; square, cytokine; diamond, enzyme; inverted triangle, kinase; circle, other; triangle, phosphatase; oval, transcription regulator; trapezoid, transporter). The network was generated through the use of IPA (QIAGEN Inc., https://www.qiagenbioinformatics.com/products/ingenuity-pathway-analysis).

**Figure 2:**
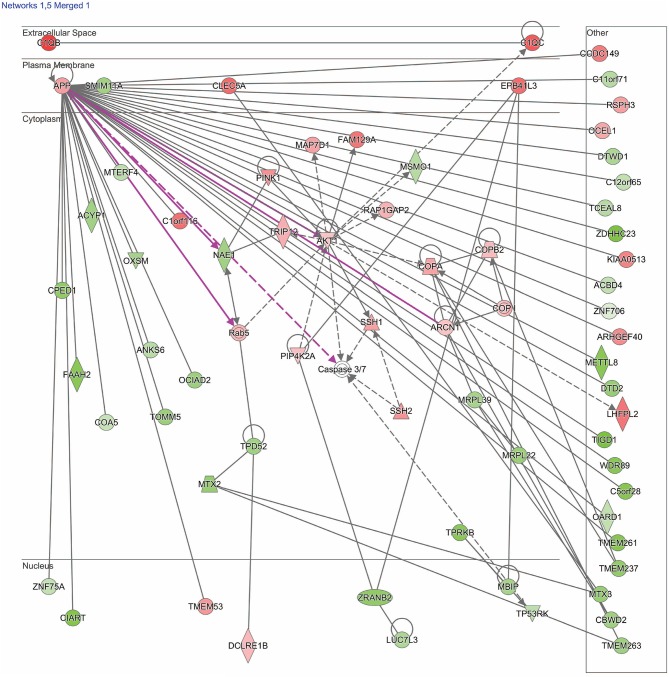
Network shows dysregulated structure, function, and development pathway following moderate blast. Genes described in the text include: *TRIP12, NAE1, AKT1, MBIP, COA5*, and *EPB41L3*. Red indicates increased measurement; green represents decreased measurement; with increased color saturation representing more extreme measurement in dataset. Solid lines represent direct interactions, non-targeting interactions, or correlations between chemicals, proteins, or RNA. Dotted lines represent indirect interactions. Purple lines denote the commonly found relationship among the two merged networks. Arrowed lines represent activation, causation, expression, localization, membership, modification, molecular cleavage, phosphorylation, protein-DNA interactions, protein-RNA interactions, regulation of binding, transcription. Shapes represent molecule type (double circle, complex/group; square, cytokine; diamond, enzyme; inverted triangle, kinase; circle, other; triangle, phosphatase; oval, transcription regulator; trapezoid, transporter). The network was generated through the use of IPA (QIAGEN Inc., https://www.qiagenbioinformatics.com/products/ingenuity-pathway-analysis).

Dysregulated genes were further explored using IPA software (build version 389077M, content version 27821452, released 2016-06-14, Qiagen, Redwood City, CA). Two pathways of interest were identified (see section Results for details and Figures 1, 2).

#### NanoString

A subset of genes examined in RNA-seq data were selected to validate gene expression changes using a direct digital detection system (NanoString Technologies, Seattle, WA). In selecting genes to validate, the extent of dysregulation, biological plausibility, and the position of the protein within the IPA pathway diagrams were considered. Two pathways were identified: one focused on cell death and survival and another focused on basic structure, function, and development. A panel was designed for each pathway to include 50 markers of interest, plus a total of nine reference genes for data normalization (Tables 2, 3). Reference genes met the following criteria: (1) not dysregulated in the RNA-seq data for the same samples; (2) not clearly implicated in traumatic brain injury, blast exposure, or a similar condition; and (4) no published evidence that this was an unstable reference gene in human blood. Probes for the 50 genes of interest and the reference genes were designed and manufactured by NanoString Technologies. NanoString was used to determine the mean copy number of each mRNA probe of interest based on manufacturer's protocol. The standard manufacturer protocol was followed for sample preparation, hybridization, and detection.

**Table 2:** Genes included in the cell death and survival pathway.

**Gene symbol**	**Gene name**	**Ref seq accession**	**HKG**	**log2 fold change**	**Adjusted *p*-value**
RPL9^*^	Ribosomal Protein L9	NM_000661.4	–	−0.714	0.002
MRPL1^*^	Mitochondrial Ribosomal Protein L1	NM_020236.3	–	−0.624	0.003
MRPS14^*^	Mitochondrial Ribosomal Protein S14	NM_022100.1	–	−0.278	0.004
MAGEH1^*^	MAGE Family Member H1	NM_014061.3	–	−0.295	0.004
BIRC3^*^	Baculoviral IAP Repeat Containing 3	NM_182962.2	–	−0.507	0.006
ARRB1^*^	Arrestin Beta 1	NM_004041.3	–	0.279	0.006
TSPYL4^*^	TSPY Like 4	NM_021648.4	–	−0.247	0.009
TESPA1^*^	Thymocyte Expressed, Positive Selection Associated 1	NM_001098815.2	–	−0.261	0.010
RPL35^*^	Ribosomal Protein L35	NM_007209.3	–	−0.431	0.011
MRPL50^*^	Mitochondrial Ribosomal Protein L50	NM_019051.1	–	−0.461	0.011
MRPL46^*^	Mitochondrial Ribosomal Protein L46	NM_022163.3	–	−0.261	0.011
ZNF32^*^	Zinc Finger Protein 32	NM_006973.2	Yes	−0.356	0.013
ZNF106^*^	Zinc Finger Protein 106	NM_022473.1	–	0.294	0.015
STAU1^*^	Staufen Double-Stranded RNA Binding Protein 1	NM_017454.2	–	0.193	0.016
TPM4^*^	Tropomyosin 4	NM_003290.2	–	0.161	0.016
RPL11^*^	Ribosomal Protein L11	NM_000975.2	–	−0.423	0.017
NSMCE4A^*^	NSE4 Homolog A, SMC5-SMC6 Complex Component	NM_017615.2	–	−0.244	0.017
RPL30^*^	Ribosomal Protein L30	NM_000989.2	–	−0.408	0.017
RPL6^*^	Ribosomal Protein L6	NM_000970.3	–	−0.340	0.018
FLT3^*^	Fms Related Tyrosine Kinase 3	NM_004119.2	–	0.390	0.019
RNF10^*^	Ring Finger Protein 10	NM_014868.3	–	0.363	0.020
RPL4^*^	Ribosomal Protein L4	NM_000968.2	–	−0.290	0.020
ACBD4^*^	Acyl-CoA Binding Domain Containing 4	NM_024722.2	–	−0.199	0.020
RPL22^*^	Ribosomal Protein L22	NM_000983.3	–	−0.265	0.021
HMGN3^*^	High Mobility Group Nucleosomal Binding Domain 3	NM_004242.3	–	−0.201	0.023
RPL15^*^	Ribosomal Protein L15	NM_001253379.1	–	−0.285	0.023
PGK1	Phosphoglycerate Kinase 1	NM_000291.2	Yes	0.194	0.024
ZBTB7B^*^	Zinc Finger And BTB Domain Containing 7B	NM_015872.2	–	0.243	0.025
ZC3H15^*^	Zinc Finger CCCH-Type Containing 15	NM_018471.2	–	−0.335	0.027
TRMT10B	TRNA Methyltransferase 10B	NM_144964.3	–	−0.197	0.027
UPF3B^*^	UPF3 Regulator Of Nonsense Transcripts Homolog B (Yeast)	NM_080632.2	–	−0.202	0.027
ALAS1	5'-Aminolevulinate Synthase 1	NM_000688.4	Yes	0.243	0.028
RFX2^*^	Regulatory Factor X2	NM_000635.3	–	0.371	0.029
PSTPIP2^*^	Proline-Serine-Threonine Phosphatase Interacting Protein 2	NM_024430.3	–	0.327	0.029
MRPL3^*^	Mitochondrial Ribosomal Protein L3	NM_007208.2	–	−0.330	0.030
ZKSCAN3^*^	Zinc Finger With KRAB And SCAN Domains 3	NM_001242895.1	–	−0.254	0.031
GUSB	Glucuronidase beta	NM_000181.3	Yes	0.194	0.034
UPF1^*^	UPF1, RNA Helicase And ATPase	NM_002911.3	–	0.255	0.036
KCNH7^*^	Potassium Voltage-Gated Channel Subfamily H Member 7	NM_033272.2	–	0.441	0.038
SH3YL1^*^	SH3 And SYLF Domain Containing 1	NM_001159597.1	–	−0.312	0.038
PARK2^*^	Parkin RBR E3 Ubiquitin Protein Ligase	NM_004562.2	–	−0.462	0.039
ALOX12B^*^	Arachidonate 12-Lipoxygenase, 12R Type	NM_001139.2	–	−0.347	0.040
NAP1L2^*^	Nucleosome Assembly Protein 1 Like 2	NM_021963.3	–	−0.394	0.041
NAP1L3^*^	Nucleosome Assembly Protein 1 Like 3	NM_004538.4	–	−0.403	0.043
DIDO1^*^	Death inducer-obliterator 1	NM_001193369.1	–	−0.093	0.045
ARAP1^*^	ArfGAP With RhoGAP Domain, Ankyrin Repeat And PH Domain 1	NM_001040118.2	–	0.270	0.045
MRPS28^*^	Mitochondrial Ribosomal Protein S28	NM_014018.2	–	−0.368	0.046
GRB2^*^	Growth Factor Receptor Bound Protein 2	NM_002086.4	–	0.214	0.048
HIBCH^*^	3-Hydroxyisobutyryl-CoA Hydrolase	NM_014362.3	–	−0.137	0.049
MOCS2^*^	Molybdenum Cofactor Synthesis 2	NM_004531.4	–	−0.267	0.049
BCL2L13^*^	BCL2 Like 13	NM_001270733.1	–	0.180	0.049
NSMCE3^*^	NSE3 Homolog, SMC5-SMC6 Complex Component	NM_138704.2	–	−0.139	0.070
ALOXE3^*^	Arachidonate Lipoxygenase 3	NM_001165960.1	–	−0.228	1.000
ABCF1	ATP Binding Cassette Subfamily F Member 1	NM_001090.2	Yes		
DECR1	2,4-Dienoyl-CoA Reductase 1, Mitochondrial	NM_001359.1	Yes		
GAPDH	Glyceraldehyde-3-Phosphate Dehydrogenase	NM_002046.3	Yes		
HPRT1	Hypoxanthine Phosphoribosyltransferase 1	NM_000194.1	Yes		
IPO8	Importin 8	NM_006390.2	Yes		
−93 miR	MicroRNA 93	NR_029510.1	Yes		
TBP	TATA-Box Binding Protein	NM_001172085.1	Yes		

* *Validated by NanoString; HKG, house-keeping gene*.

**Table 3:** Genes included in the structure, function, and development pathway.

**Gene symbol**	**Gene name**	**Ref Seq accession**	**HKG**	**log2 fold change**	**Adjusted *p*-value**
LHFPL2^*^	Lipoma HMGIC Fusion Partner-Like 2	NM_005779.2	–	0.401	0.001
TMEM261^*^	Transmembrane Protein 261	NM_001318058.1	–	−0.465	0.003
TRIP12^*^	Thyroid Hormone Receptor Interactor 12	NM_004238.1	–	0.231	0.003
ZDHHC23	Zinc Finger CCHC-Type Containing 23	NM_173570.3	–	−0.521	0.004
OCIAD2	Ovarian Carcinoma Immunoreactive Antigen-Like Protein 2	NM_152398.2	–	−0.350	0.005
FAAH2	Fatty Acid Amide Hydrolase 2	NM_174912.3	–	−0.499	0.006
TPRKB^*^	TP53RK Binding Protein	NM_016058.2	–	−0.443	0.006
EPB41L3^*^	Erythrocyte Membrane Protein Band 4.1 Like 3	NM_012307.2	–	0.431	0.006
NAE1^*^	NEDD8 Activating Enzyme E1 Subunit 1	NM_001018159.1	–	−0.324	0.007
SSH1^*^	Slingshot Protein Phosphatase 1	NM_018984.3	–	0.269	0.010
RAP1GAP2^*^	RAP1 GTPase Activating Protein 2	NM_015085.4	–	0.219	0.011
C1QB^*^	Complement Component 1, Q Subcomponent, B Chain	NM_000491.3	–	0.584	0.011
OARD1^*^	O-Acyl-ADP-Ribose Deacylase 1	NM_145063.2	–	−0.205	0.012
COPB2^*^	Coatamer Protein Complex Subunit Beta	NM_004766.2	–	0.193	0.012
TIGD1^*^	Tigger Transposable Element Derived 1	NM_145702.1	–	−0.531	0.012
CLEC5A^*^	C-Type Lectin Domain Family 5 Member A	NM_013252.2	–	0.437	0.014
KIAA0513^*^	KIAA0513 Ortholog	NM_014732.3	–	0.361	0.015
MRPL39^*^	Mitochondrial Ribosomal Protein L39	NM_017446.3	–	−0.304	0.015
DCLRE1B^*^	DNA Cross-Link Repair 1B	NM_022836.3	–	0.198	0.017
MBIP^*^	MAP3K12 Binding Inhibitory Protein 1	NM_001144891.1	–	−0.305	0.017
TP53RK^*^	TP53 Regulating Kinase	NM_033550.3	–	−0.213	0.018
COPA^*^	Coatamer Protein Complex Subunit Alpha	NM_004371.3	–	0.259	0.019
ACYP1^*^	Acylphosphatase 1	NM_001107.3	–	−0.329	0.019
ZRANB2^*^	Zinc Finger RANBP2-Type Containing 2	NM_005455.4	–	−0.404	0.019
MTX2^*^	Metaxin2	NM_006554.4	–	−0.369	0.020
ABCD4^*^	ATP Binding Cassette Subfamily D Member 4	NR_003256.2	–	−0.199	0.020
MRPL22^*^	Mitochondrial Ribosomal Protein L22	NM_014180.3	–	−0.361	0.023
PGK1	Phosphoglycerate Kinase 1	NM_000291.2	Yes	0.194	0.024
SSH2^*^	Slingshot Protein Phosphatase 2	NM_033389.3	–	0.315	0.024
FAM129A^*^	Family with sequence similarity 129, member A	NM_052966.2	–	0.403	0.025
C12orf65^*^	Chromosome 12 open reading frame 65	NM_152269.4	–	−0.204	0.026
ALAS1	5'-Aminolevulinate Synthase 1	NM_000688.4	Yes	0.243	0.028
OXSM^*^	3-Oxoacyl- Acyl Carrier Protein Synthase, Mitochondrial	NM_017897.2	–	−0.306	0.028
COA5^*^	Cytochrome C Oxidase Assembly Factor 5	NM_001008215.2	–	−0.188	0.030
ZNF706^*^	Zinc finger protein 706	NM_001042510.1	–	−0.109	0.031
RSPH3^*^	Radial Spoke 3 Homolog	NM_031924.4	–	0.272	0.032
TMEM237^*^	Transmembrane Protein 237	NM_001044385.1	–	−0.394	0.032
MTX3^*^	Metaxin3	NM_001010891.4	–	−0.370	0.033
PIP4K2A^*^	Phosphatidylinositol-5-Phosphate 4-Kinase Type 2 Alpha	NM_005028.3	–	0.181	0.033
APP^*^	Amyloid Precursor Protein	NM_000484.3	–	0.275	0.034
GUSB	Glucuronidase Beta	NM_000181.3	Yes	0.194	0.034
TPD52^*^	Tumor Protein D52	NM_005079.2	–	−0.330	0.038
CIART^*^	Circadian Associated Repressor of Transcription	NM_144697.2	–	−0.475	0.038
AKT1^*^	AKT Serine/Threonine Kinase 1	NM_001014432.1	–	0.170	0.039
MAP7D1^*^	MAP7 Domain Containing 1	NM_018067.3	–	0.267	0.040
ANKS6^*^	Ankyrin Repeat and Sterile Alpha Motif Domain Containing 6	NM_173551.3	–	−0.236	0.041
MSMO1^*^	Methylsterol Monooxygenase 1	NM_001017369.1	–	−0.238	0.041
LUC7L3^*^	LUC7 Like 3 Pre-MRNA Splicing Factor	NM_006107.2	–	−0.279	0.042
TCEAL8^*^	Transcription Elongation Factor A Like 8	NM_153333.2	–	−0.243	0.045
TOMM5^*^	Translocase Of Outer Mitochondrial Membrane 5	NM_001001790.2	–	−0.354	0.046
ARCN1^*^	Archain 1	NM_001655.4	–	0.171	0.047
TMEM263	Transmembrane Protein 263	NM_152261.2	–	−0.342	0.049
RAB5A^*^	RAS-Associated Protein RAB5A	NM_004162.4	–	0.182	0.136
ABCF1	ATP Binding Cassette Subfamily F Member 1	NM_001090.2	Yes		
DECR1	2,4-Dienoyl-CoA Reductase 1	NM_001359.1	Yes		
GAPDH	Glyceraldehyde-3-Phosphate Dehydrogenase	NM_002046.3	Yes		
HPRT1	Hypoxanthine Phosphoribosyltransferase 1	NM_000194.1	Yes		
IPO8	Importin 8	NM_006390.2	Yes		
miR-93		NR_029510.1	Yes		
TBP	TATA-Box Binding Protein	NM_001172085.1	Yes		

* *NanoString validation; HKG, house-keeping gene*.

## Statistical Analysis

### Overview

The Statistical Package for the Social Sciences (SPSS; version 22; IBM Corporation, Armonk, NY) and NanoString's nSolver Analysis Software (version 3; NanoString Technologies, Seattle, WA) were usedfor analyses.

### RNA-Seq Analysis

The moderate blast exposed cases (*n* = 29) met QC criteria based on the RNA Integrity Number (RIN) and were subsequently sequenced. In total, between 52.5 and 75.5 million read counts were completed for each sample; in 94.95% of base calls, an accuracy of at least Q30 was achieved. To establish bioinformatics quality control (QC), FastQC (version 0.11.5, Babraham Bioinformatics, Cambridgeshire, UK) was used. Data were aligned to a reference genome (hg19) using an open-source aligner, STAR (version 2.5) (30). To count the number of reads mapped to genes, HTSeq software was used (version 0.6.1p1) (31). DESeq2 (version 1.12.3) (32) was used to identify differentially expressed genes, with cutoff value of false discovery rate (FDR) of 0.05. This cut off value of the false discovery rate is considered to be a conservative balance in minimizing both false positives (type I errors) and false negatives (type II errors) (33, 34).

### NanoString Validation Analysis

RNA sequencing data were validated with 50 genes from each network (100 genes total), plus reference genes, using a custom NanoString codeset. Raw data were analyzed using nSolver 3.0 digital analyzer software using standard settings and quality control parameters. Raw data were normalized against reference genes. Gene expression differences before and after blast exposure were measured using *t*-tests, with the Benjamini-Yekutieli FDR method for multiple comparison corrections to conservatively balance the minimization of type I and type II errors (35). Log_2_ fold changes and adjusted *p*-values were calculated for samples before and after blast exposure, with statistical significance defined at the level of *p* < 0.05, demonstrating congruency with RNA sequencing findings.

## Results

### Demographic Results

Participants in the study were male military service members with a mean age of 30.42 and a mean length of service of 9.94 years (Table 1). Almost half of participants (46.3%) had a history of >40 prior blast exposures. No significant differences based on demographic information were noted among the cohort [described in previous report by Gill et al. (13)].

### RNA-Seq Results

Results of the RNA-seq analysis demonstrated significantly dysregulated gene activity changes following moderate blast exposure. These genes were entered into IPA software (29). IPA analysis identified five significantly dysregulated networks before and after moderate blast exposure (Table 4). Two sets of two networks shared overlapping functions and were subsequently merged to form two networks of interest in the present study: (1) Cell Death and Survival and (2) Cell Structure, Function, and Metabolism (Table 4). NanoString analysis confirmed 32 significantly differentially expressed genes in the Cell Death and Survival network (*p* < 0.05) and 35 significantly differentially expressed genes in the Cell Structure, Function, and Metabolism network (*p* < 0.05), validating differential expression of these two gene networks following blast exposure. Here, we report on the most significant gene network activity changes in the Cell Death and Survival and the Cell Structure, Function, and Metabolism networks (Figures 1, 2).

**Table 4:** IPA Network Scores.

**Network**	**IPA network score**
Metabolic	45
Cell death and survival	42
Post-translational modification	42
Cancer, cell death and survival	42
Immunological diseases	37
**Merged networks**	
Cell death and survival	42
Cell structure, function, and metabolism	41

*Network scores are numerical values used to rank fit of molecules to the network. The scores are calculated using an algorithm based on Fisher's Exact Test. Eligible molecules are compared to the Ingenuity Knowledge Base of over 1 million molecules curated from literature findings. Highly interconnected genes imply significant biological function*.

#### Cell Death and Survival Network

One merged pathway centered on cell death and survival. Genes identified in this pathway are defined in Table 2. This pathway was comprised of genes implicated in apoptosis, necrosis, autophagy, mitophagy, ferroptosis, survival, regeneration, and recovery, with an IPA score of 42 (Figure 1). Significantly dysregulated genes include UPF1, RNA helicase and ATPase *(UPF1)*, UPF3 regulator of nonsense transcripts homolog B (*UPF3B*), arrestin β1 (*ARRB1*), zinc finger and BTB domain containing 7B (*ZBTB7B*), fms related tyrosine kinase 3 (*flt3*), 3-hydroxyisobutyryl-CoA hydrolase (*HIBCH*), ribosomal proteins (*RPL6, -L35*), and mitochondrial ribosomal proteins (*MRPL1, -L3, -L36*, and -*L50*). Major hubs within this network include growth factor receptor bound protein 2 (*GRB2*) and staufen double-stranded RNA binding protein 1 (*STAU1*).

#### Cell Structure, Function, and Metabolism Network

The second merged pathway focused on development, metabolism, and cell structure/function. Genes identified in this pathway are defined in Table 3. This pathway consisted of genes involved in cytoskeleton, organelles, cellular metabolism, lipid metabolism, heat shock, cell motion, cell growth, and differentiation, with an IPA score of 41 (Figure 2). Significant genes within this network include tripartite motif containing 12 (*TRIP12)*, NEDD8 activating enzyme E1 subunit 1 (*NAE1*), cytochrome C oxidase assembly factor 5 (*COA5*), and erythrocyte membrane protein band 4.1-like 3 (*EPB41L3*). AKT serine/threonine kinase 1 (*AKT1*), amyloid precursor protein (*APP*), and MAP3K12 binding inhibitory protein 1 (*MBIP*) are the major hubs in this network.

## Discussion

Activity changes in two gene networks were found after moderate blast exposure in military personnel engaged in training. Differentially regulated networks after blast included (1) Cell Death and Survival (Figure 1) and (2) Cell Structure, Function, and Development (Figure 2). Genes within these two networks relate to ubiquitination, nonsense mediated decay (NMD), apoptosis, as well as activity related to ribosomes, mitochondria, and inflammation. Findings provide novel insights for understanding the biological changes that occur following blast, which for some individuals, may result in biological changes that increase their risk for neurological or behavioral symptoms and deficits. These findings may ultimately contribute to characterizing the cellular mechanisms of blast exposure to improve diagnosis, monitoring, and prognosis of military personnel exposed to blast.

In this study, genes related to ubiquitination were increased in activity following blast exposure, including tripartite motif containing 12 (*TRIP12)*, which is an E3 ubiquitin-protein ligase involved in ubiquitin fusion degradation. Protein ubiquitination initiates the removal of oxidized and misfolded proteins following injury, and its processes can protect neurons from reactive oxidative species (ROS) that accumulate following blast exposure in pre-clinical models (36). Our findings provide further evidence of increased UCH-L1, the primary protein for ubiquitination, following repeated low-level blast (12). This finding suggests that there may also be overlap with the biological mechanisms related to recovery from TBIs in civilians, as UCHL1 increases are one of the most often reported changes following a TBI (37, 38). However, these findings are in contrast to another previous report in which the activity of genes related to ubiquitin were lower in activity in military personnel with TBIs, with many related to blast exposures, and chronic symptoms (11). Therefore, it may be that ubiquitin activity is critical to acute recovery from biTBIs, and that in some individuals, there is a reduction in activity that may place them at higher risk for chronic symptoms. In support of this, pre-clinical studies show that reductions or inactivation of ubiquitin activity results in poor outcomes, including behavioral deficits, possibly indicating long-term neurodegenerative processes (39).

Genes that may relate to neuronal recovery were altered in activity following a moderate blast. Specifically, we report gene activity changes within the NMD pathway, including *UPF1* and *UPF3B*, which are responsible for neuronal specific cell development and repair through a reciprocal pattern of activity (40). Previous studies show an interaction in the activity of these two genes, such that when one gene is less active, the other gene will compensate, preserving the activity of this network; our findings mirror this. Here we report that *UPF1* was increased in activity, whereas *UPF3B* was downregulated. These findings suggest that in response to the blast, injury mechanisms may have been initiated (inflammation, aberrant cellular formation, and cell death), and this initiation may result in an upregulation of *UPF1*, in an effort to preserve the activity of the NMD pathway. Subsequently, here we report that the expression of *UPF3B* is suppressed, hindering possible detrimental neurological effects. These findings suggest complex gene-activity changes following blast exposure that may be occurring to promote recovery; additional studies are needed to increase understanding of the temporal relationship of these changes and their relation to neuronal recovery.

A downregulated gene within the structure, function, and development pathway was *NAE1* (NEDD8 Activating Enzyme E1 Subunit 1), a protein associated with the neddylation pathway. Vogl et al. showed that neddylation was a critical regulator of dendritic spine development, reporting that in *NAE1* knockout mice, there were cognitive deficits as well as synaptic and neurotransmitter impairments (41). The down regulation observed in our military population could suggest similarly that exposure to blast hinders the neddylation pathway, which might suggest a marker of injury resulting directly from blast exposure. Additionally, recent *in vitro* work suggests Il-1β may inhibit NEDD8 and neddylation in conjunction with increased ubiquitination; while activation of NEDD8 downregulates the nuclear factor kappa light-chain enhancer of activated B cells (NF-κB) pathway (42). This finding is of interest because we also found that genes within the NF-κB network show activity changes, with most genes becoming more active. The NF-κB network is a dominant activator of the immune system following TBI, and this activity is essential because it initiates secondary injury mechanisms required for neuronal recovery. However, if activity of this pathway is too high, or too long-lasting, it can be detrimental to neuronal recovery (43). One such gene is *ARRB1*(arrestin β1), which is increased following blast exposure. This gene has been reported to play a role in the beta-adrenergic receptor kinase (BARK) mediated desensitization of beta-adrenergic receptors. In TBI patients, catecholamine surge after injury has been linked to immunosuppression and greater mortality risk that is reversed through β-blocker treatment (44). *ZBTB7B* (zinc finger and BTB domain containing 7B) is also upregulated after blast and linked to reductions in CD8-cytotoxic activity (45), which could be a mechanism to prevent further cellular damage after blast injury.

In addition to the activation of genes within the NF-κB network, *AKT1* is another immune-related gene with increased activity. *AKT1* is a hub with 14 connections in the structure, function, and development network. *AKT1* encodes for a serine-threonine protein kinase (AKT1), which is known to regulate a vast number of cellular processes including neuronal survival, glucose uptake, protein and fatty acid synthesis, cell proliferation, and the previously mentioned role in apoptosis (46, 47). Additionally, *AKT1* may function in the inflammatory response as an upstream activator of NF-κB (48). Interestingly, in this population, significantly elevated levels of the cytokines tumor necrosis factor alpha (TNFα) and interleukin 6 (IL-6) have been reported during the acute period following moderate blast (49). This finding is relevant because NF-κB is recognized as a master regulator of cytokines including TNFα and IL-6 (47, 50). The NF-κB pathway has been implicated in the regulation of proinflammatory cytokines during meningitis (51) and in blood-brain barrier permeability (52). Moreover, the NF-κB pathway has been found to be dysregulated in clinical studies of acute and subacute TBI (7–10). Upregulation of *AKT1* in this sample suggests activation of the NF-κB pathway; a finding that supports these prior studies, although the specific role of *AKT1* in blast effects on the central nervous system remains to be examined.

Also showing increased activity in this study was the *Flt3* (dimer), which encodes for a receptor tyrosine kinase and is also related to NF-κB pathway. *Flt3* is implicated in multiple signaling pathways including regulation of the proliferation and survival of hematopoietic cells, which ultimately relates to the number of intermediate monocytes (53). Because intermediate monocytes promote production of inflammatory cytokines within the NF-κb network, including TNF-α and Il-1β (54), these findings suggest the possibility of a pro-inflammatory response through increased production of intermediate monocytes.

Our findings of changes in activity of apoptosis-related genes following blast are of interest because preclinical models show that blast exposure results in astrocytic and microglial activation, oxidative stress, axonal and vascular damage, and inflammation, which ultimately contribute to programmed cell-death (55–58). Specifically, we found activation of caspase complexes, a family of cysteine-dependent proteases, which have been previously associated with neuronal and oligo-dendroglial cell death in both pre-clinical and human brain injuries (59). Otherwise referred to as apoptosis executioners, caspase-3 and -7 are both indirectly activated by *MBIP*, a major hub of the cell structure, function, and metabolism network. Increased expression in caspase-3 and -7 complexes have also been previously linked to TBIs in pre-clinical models (60, 61) and to mortality in patients with severe TBIs (62). We also report increased activity of other apoptosis genes following blast including *EPB41L3*, or erythrocyte membrane protein band 4.1-like 3, and *EPB41L3*, a tumor suppressor gene strongly expressed in the brain that promotes apoptotic pathways and inhibits cellular proliferation (63). These findings suggest that moderate blast results in expression of apoptosis inducing genes, and that mitigating these activities may be protective.

Lastly, several mitochondrial genes and genes connected with the mitochondrial gene network are dysregulated, including *COA5, HIBCH, RPL6, RPL35*, as well as mitochondrial ribosomal genes *MRPL50, MRPL1, MRPL3*, and *MRPL46*. Although the function of mitochondria is not yet well-understood in blast exposures, it is worth noting that mitochondrial dysfunction has been implicated in preclinical TBI pathology. Previous studies have indicated that following TBI an influx of intracellular calcium leads to disruption of the mitochondrial membrane potential, impairing ATP production and creating ROS, activating cell death pathways and leading to neuronal damage associated with cognitive impairments (64, 65). The biological mechanisms specific to blast effects on the central nervous system in the context of mitochondrial genes are not yet known.

Alterations in these gene expression pathways may have translational implications for blast-related neuropathology if replicated. In this military training population, dysregulation in gene expression pathways related to ubiquitination, NMD, apoptosis, inflammation, and ribosomal and mitochondrial activity were observed, suggesting a role for these pathways following acute blast exposures. Examination of related, downstream proteins may indicate potential diagnostic or prognostic biomarkers. Mapping temporal changes in gene expression, downstream proteins, and symptomology may shed light on the role of these pathways in underlying neuropathological processes and clinical outcomes.

### Limitations

Although these initial findings provide novel insights into gene-activity changes following blast, this study has a number of limitations. First, the secondary data analysis in this study precluded the comparison of control personnel who were engaged in blast training, but did not sustain a moderate blast; therefore, gene expression changes related to normal daily training activities or other activities of daily living such as circadian rhythm or diet cannot be determined. In addition, a sub-portion of the moderate blast cohort were experienced trainers with previous blast exposures, and thus were not naïve to blast. However, this sample represents typical training cohorts. Second, blast exposure may affect cell types throughout the body in addition to brain-related pathways. Preclinical models demonstrate that the CNS is affected by blast exposure (66, 67); however, at this time translating this information to clinical populations presents difficulty as accessing the CNS requires intensive, invasive procedures. Third, due to the exploratory nature of this work, clinical symptomology measures were not collected.

These limitations represent important considerations for future work. Additional studies in clinical blast exposure will need to collect samples from unaffected control cohorts, not only a priori matched samples, in order to differentiate possible causative and confounding agents such as blast exposure vs. training effects. We are addressing this design limitation in future studies. Next, considering the limitation in directly studying the CNS in clinical populations, an exciting future direction for this work would be the ability to measure brain-specific peripheral biomarkers, in order to differentiate brain-related pathways from other cellular processes influenced by blast exposures. Finally, the significant gene expression changes found in this study warrant further research into possible symptomology that may be experienced following blast exposure utilizing validated clinical measures and mapping these symptoms to biomarker changes. Notably, this study identified altered gene expression pathways, which may be the focus of more specific biological mechanisms significant to blast exposure in future studies.

## Conclusion

The initial findings reported here show that there are robust gene activity changes following a moderate blast exposure in a sample of military personnel. Notably, this study's findings are important to distinguish the effects of blast exposure without the co-occurring impact of blunt-force injuries that take place in combat stations. Findings from this study suggest that additional studies are needed to examine gene-activity related to blast exposure, in order to examine the impact of previous blast exposures on biomarker changes, relationships to neurological impacts, and subsequent clinical symptoms. Thus, these findings provide novel insights into gene network dysregulation observed following objectively measured blast exposure that warrant future clinical studies to advance the understanding of neuropathology related to blast exposure.

## Data Availability Statement

Sequencing data used in the study were deposited in the Gene Expression Omnibus (GEO) with GEO ID GSE89866.

## Ethics Statement

This study protocol was reviewed and approved by the Institutional Review Boards at the Naval Medical Research Center and Walter Reed Army Institute of Research (NMRC#2011.0002; WRAIR#1796). All subjects gave written informed consent prior to participation in the study.

## Author Contributions

KE, JG, NO, and H-SK contributed to the conception or design of the study. KE, VM, SY, Y-EC, and CL contributed to lab analysis, interpretation of the data, and drafting of the work. KE, SY, Y-EC, CL, KD, WC, PW, SA, ML, AY, AT, and JG contributed to the collection, interpretation of the data, and drafting and editing of the work. All authors contributed to critical revision of the manuscript, read and approved the submitted version, and agree to be accountable for all aspects of the work in ensuring that questions related to the accuracy or integrity of any part of the work are appropriately investigated and resolved.

### Conflict of Interest

The authors declare that the research was conducted in the absence of any commercial or financial relationships that could be construed as a potential conflict of interest. The opinions and assertions in this manuscript are those of the authors and are not to be construed as official as reflecting true views of the Department of Navy, Department of the Army, Department of Defense, the Uniformed Services University of the Health Sciences, U.S. Government, the Center for Neuroscience and Regenerative Medicine or any other agency of the U.S. government. This study was funded by National Institutes of Health, Intramural Department of Research, and the US Army Medical Research and Material Command and the US Navy Bureau of Medicine (NMRC#2011.0002; WRAIR#1796). The study protocol was approved by the Walter Reed Army Institute of Research/Naval Medical Research Center Institutional Review Boards in compliance with AR 70-25 and all applicable Federal regulations governing the protection of human subjects. Some of the authors are military service members or employees of the U.S. Government. This work was prepared as part of their official duties. Title 17 U.S.C. §105 provides that “Copyright protection under this title is not available for any work of the United States Government.” Title 17 U.S.C. §101 defines a U.S. Government work as a work prepared by a military service member or employee of the U.S. Government as part of that person's official duties. Material has been reviewed by the Walter Reed Army Institute of Research. There is no objection to its presentation and/or publication.
